# Variation analysis and comparison of leaf and fruit traits of triploid hybrid progeny in jujube

**DOI:** 10.3389/fpls.2025.1553316

**Published:** 2025-03-07

**Authors:** Jiayuan Xuan, Quanhui Ma, Lixin Ge, Fenfen Yan, Jun Yu, Jiurui Wang, Cuiyun Wu, Mengjun Liu

**Affiliations:** ^1^ College of Horticulture and Forestry, Tarim University/The National and Local Joint Engineering Laboratory of High Efficiency and Superior-Quality Cultivation and Fruit Deep Processing Technology of Characteristic Fruit Trees in Southern Xinjiang, Alar, China; ^2^ Xinjiang Production and Construction Crops Key Laboratory of Protection and Utilization of Biological Resources in Tarim Basin, Tarim University, Alar, China; ^3^ College of Forestry, Hebei Agricultural University, Baoding, China; ^4^ College of Horticulture, Hebei Agricultural University, Baoding, China

**Keywords:** jujube, triploid, hybrid progeny, leaves, fruits, variation analysis

## Abstract

**Introduction:**

Polyploid hybrid progeny have both ploidy and hybridization effect, and abound in genetic variation of traits, which is an important material for the breeding of excellent new varieties.Ploidy breeding is an important means to improve and cultivate new varieties, which could overcome distant hybridization incompatibility and play a great significance to species evolution and environmental adaptation.Therefore, triploid breeding has a wide range of application value in germplasm innovation and cultivation of excellent new varieties of fruit trees.

**Methods:**

In order to reveal the trait characteristics and variation analysis of leaves and fruits of triploid hybrids, the ploidy identifications and SSR analysis were carried out through all progeny, and the leaf, stomata, thorn, fruit quality and other traits were compared and analyzed between the triploid and diploid progeny. The genetic variation of the triploid progeny were further analyzed, which could provide reference for hybrid parents selection, offspring traits prediction, ploidy breeding of jujube.

**Results:**

The results indicated that the triploid progeny exhibited significantly higher values for leaf width, straight thorn length, and stomatal length compared to diploid individuals. However, the leaf shape index and stomatal density were significantly lower in triploids than in diploids. Furthermore, a low genetic trend was observed for leaf traits in triploid progeny, while needle prick traits displayed a high genetic trend. In contrast, stomatal width and stomatal density showed moderate to low genetic trends. Significantly, the single fruit weight, single fruit kernel weight and soluble solids content were significantly higher in triploids compared to diploids. However, the kernel index and titratable acid content were significantly lower in triploids than diploids. The genetic trend showed a high inclination towards single fruit weight, fruit length, fruit shape index, kernel transverse diameter and vitamin C content in triploid offspring. The edible rate exhibited a moderate genetic trend, while kernel longitudinal diameter, kernel index, and titratable acid content displayed a low genetic trend.

**Discussion:**

First, the occurrence of polypoloid hybrid progeny. Second, triploid hybrid progeny of jujube has typical polypolid traits. Finally, abondance of genetic variations of traits in triploid progeny.

## 1 Introduction

Jujube (*Ziziphus jujuba* Mill.) is native to China, which is an important fruit tree and the largest cultivated species in the world with the highest economic and ecological values (Liu M. J. et al., 2010). Jujube is increasingly favored and recognized by consumers because of its rich nutritional components and medicinal value (Lu et al., 2010). In 2020, the area of jujube in Xinjiang was 4.45 million Mu, and the yield was 3.8124 million tons, accounting for about 52% of China (Guo et al., 2023). Jujube industry is not only an important pillar industry of agricultural development in Xinjiang, but also an important way for farmers to increase their income. Jujube has the characteristics of resistance to drought, barren soil, salt and alkali stresses, but less tolerance to cold stress (Qi et al., 2024; Wang et al., 2023). It plays an important role in ecological construction, agricultural industrial structure adjustment, returning farmland to forest, sand prevention, wind and sand fixation in arid areas of northwest China such as Xinjiang (Wu et al., 2016). At present, the development of Xinjiang jujube industry faced several challenges, such as unreasonable variety structure, lack of special processing varieties, imbalance of early, middle and late maturing varieties (Li and Wei, 2013). In the process of cross breeding of jujube, due to the important limitation of small flowers, low fruit setting rate and high embryo abortion rate (Liu et al., 2013), the hybridization efficiency is low and the breeding process of new varieties is slow. Therefore, finding an efficient and accurate breeding method can effectively solve the current dilemma of jujube breeding and promote the creation of new jujube germplasms.

Ploidy breeding is an important means to improve and cultivate new varieties, which could overcome distant hybridization incompatibility and play a great significance to species evolution and environmental adaptation. Excellent polyploid germplasms has been created by sexual polyploidization means in different fruit tree species, including apple, pear, grape, plum, kiwi, etc (Wang et al., 2004). Hybridization between different ploidy is an important way to create polyploid progeny. For example diploid seedless grapes and tetraploid Kyoho hybridization was applied to obtain triploid grape offsprings (Wang S. Y. et al., 2014). The triploid citrus offsprings were obtained by hybridization of polyembryony diploid and tetraploid citrus (Xie et al., 2014). The triploid ‘Huaxing’ pear was obtained by crossing tetraploid ‘Dayali’ with diploid ‘Xuehua’ (Wang F. et al., 2014). Triploid apple ‘Jonagold’, tetraploid grape ‘Kyoho’, and nine-ploid persimmon flat-core cultivars were cross bred by ploidy breeding (Pu et al., 2013). Fruit polyploid germplasm has significant advantages of huge organs, no nuclei, strong stress resistance and high metabolite content, and is also an excellent material for studying the relationship between genome doubling and plant reproduction and growth. In recent years, the citrus team of Huazhong Agricultural University has obtained a large number of new citrus triploid karyofree germplasm by using the hybrid combination of monoembryonic diploid citrus varieties and tetraploid somatic hybrids as parents (Xie et al., 2013). Zhao et al. (2006) studied the flowering characteristics of wild plants related to sweetpotato and found that tetraploid *I.littoralis* had more flowering numbers than hexploid *I.rifida*. In terms of physiology, the polyploid sweetpotato group plants also show certain differences in seed setting rate. Du (2021) studied Epigenetic regulation mechanism of vegetative growth advantage in allotriploid *Populus* spp. (Section Tacamahaca) showed that the expression of differential genes in the triploid of *populus* spp. resulted in rapid accumulation of auxin and cytokinin, which were positively related to vegetative growth, and decreased the contents of ethylene, abscisic acid and salicylic acid, which were negatively related to vegetative growth, resulting in accelerated cell division and growth, larger leaves, and increased chlorophyll content in the triploid leaves of *populus* spp. The chlorophyll degradation is slowed down, the photocontracting ability is enhanced, and more sucrose and starch can be accumulated, which has obvious growth advantages. By using diploid and tetraploid hybridization to create triploid germplasm, breeders have cultivated many new triploid varieties of apple, pear, grape, citrus, etc. Therefore, triploid breeding has a wide range of application value in germplasm innovation and cultivation of excellent new varieties of fruit trees.

Morphological traits evaluation is the most effective means to study plant genetic diversity, and the results of morphological traits can reflect the phenotypic variation of different materials (Gao, 2011). Xiang et al. (2019) determined the morphological traits of grape tetraploid progeny, which found that the coefficient of variation of 14 traits was higher than 20%. The separation interval of the maximum and minimum of the traits was in a large range, and there was a phenomenon of hyperparental separation. Xiao (2013) proposed that jujube germplasm in Jinshan area of the Yellow River Basin had high inter-population and intra-population variation and rich genetic diversity. The fruit size and sugar traits of citrus triploid hybrid progeny were quantitative traits controlled by multiple genes, and the acid traits might be regulated by main control genes. The fruit sugar and acid content of triploid citrus group which was derived from ploid hybridization tended to the high acid and low sugar parents, and was significantly impacted by the male parent (Guan et al., 2024; Wang et al., 2022).The fruit size of the hybrid progeny of tetraploid ‘Gala’ and diploid ‘Tengmu No.1’ showed a high-parent genetic trend of large fruit type, titratable acid showed a low-parent genetic trend of low acid, and fruit hardness showed a low-parent genetic trend of low hardness. The orthogonal group of fruit soluble sugar showed a high-parent genetic trend of high sugar, and the reverse cross population of fruit soluble sugar showed a low-parent genetic trend of low sugar. Overall, the reciprocal hybrids showed a genetic trend of separation in fruit flavor, with the majority of sour-sweet types (Yu, 2022). The triploid hybrid lines of diploid ‘Hanfu’ and tetraploid ‘Gala’ had a maternal genetic tendency (He et al., 2015). It is evident that polyploidy progeny represents rich trait variation and transgressive inheritance. Therefore, ploidy breeding and genetic mechanism of polyploid plant traits variation has been widely concerned by fruits breeding researchers.

Jujube germplasm resources are abundant in China, and there are 944 known varieties and excellent lines (Liu and Wang, 2009). However, only two varieties ‘Zanhuangdazao’ (Peng et al., 2005) and ‘Pingguozao’ (Liu et al., 2013) were identified as natural triploid varieties currently. Ploidy hybridization is the most simple and effective way to obtain new polyploids, but polyploid breeding of jujube is still dominated by colchicine homologous induction. Liu et al. (2010) induced the stem tip of ‘Linyilizao’ (2x) through colchicine mutagenesis, and resulting in the production of the first tetraploid jujube variety ‘Chenguang’. Thus, these researchers successfully obtained the world’ first tetraploid jujube variety ‘Chenguang. Yan et al. (2018) created 3 triploid and 1 diploid germplasms by using ‘Chenguang’ as a parent firstly. The above materials have important application values in ploidy breeding research and provide the better material support for developing new jujube polyploid varieties. The previous studies found that there are rich and popular variation in the traits of polyploid materials such as stem diameter, large leaves, large flowers and fruits (Wang et al., 2022). Luckily, more triploid and diploid germplasms were created by the hybridization of ‘Dongzao’×’Chenguang’ in the current study. In order to reveal the trait characteristics and variation analysis of leaves and fruits of triploid hybrids, the ploidy identifications and SSR analysis were carried out through all progeny, and the leaf, stomata, thorn, fruit quality and other traits were compared and analyzed between the triploid and diploid progeny. The genetic variation of the triploid progeny were further analyzed, which could provide reference for hybrid parents selection, offspring traits prediction, ploidy breeding of jujube.

## 2 Materials and methods

### 2.1 Materials

The female parent was diploid ‘Dongzao’ (DZ, 2x=24) and the male parent was tetraploid ‘Chenguang’ (CG, 4x=48). For two consecutive years, controlled hybridization was carried out through pollination by bees in nets (Qiu et al., 2022). The nets were made of thin, breathable white mosquito net gauze, with 10 to 12 plants in each cage. At the early flowering stage (mid-late May), beehives were placed in the net to raise young bees for assisting pollination. A box of 2 spleen bees were placed in each net cage, and the bees were fed with fresh water and sugar water every other week. In 2016-2017, the harvested seeds were sown in the No.4 greenhouse of the Horticultural Experimental Station of Tarim University and grew to seedlings. In 2021, the hybrid offsprings and parent scions were collected and grafted in the 12th jujube breeding test base of the First Division in Alar City, Xinjiang. The grafting rootstock is 6-year ‘Huizao’. The plants were cultivated with sufficient water, fertilizer and high consistent managements.

In 2021, 124 progeny were used to identify ploidy and hybrids. Among them, 68 were triploid progeny and 56 were diploid progeny. However, due to salinity and freezing damage in Xinjiang, some test materials are missing. Therefore, 54 triploid progeny and 16 diploid progeny with good fruit bearing and stable traits were selected as experimental materials.

### 2.2 Methods

#### 2.2.1 Flow cytometry identification

Ploidy identification was based on the method of Wang et al. (2018), with partially improvements. During the early June 2021, the tender leaves of the hybrid offsprings were collected. 0.2 g of tender leaves were taken, washed with distilled water to remove the surface dust, and then dried with filter paper and placed in a pre-cooled (4°C) petri dish. 2 mL cell lysate was added and chopped with a sharp blade to fully extract the complete nucleus. The extraction time was 60 s. Lcell lysate was extracted from the petri dish, filtered with the help of 400 mesh filter membrane into sample tube and then 200 μL of DAPI dye was added. After mixing, the sample tubes were placed in a refrigerator at 4°C and dye for 30 min in dark. After staining, the samples were analyzed by using by BD FACSCalibur flow cytometry and samples were placed in upper sample tubes for further testing and then 5,000 to 10,000 nuclei were collected. The diploid ‘Dongzao’ and tetraploid ‘Chenguang’ were used as the control. The DNA curve was generated directly by the instrument, and the ploidy of the hybrid progeny was preliminarily determined based on the measurement introduction.

#### 2.2.2 SSR identification

Genomic DNA was extracted by modified CTAB method (Xiao, 2014). In the early June 2021, the tender leaves of each testing material were collected. The SSR primers were developed based on jujube genome sequencing data through literature searching, and 5 primers with optimum polymorphism and high repeatability were screened for genetic diversity analysis and PCR amplification (**Supplementray Table 1**). The PCR reaction system was 12.5 μL, including genomic DNA 0.5 μL (about 10-25 ng), Taq Master Mix 6.25 μL, dd H2O 4.75 μL. Both forward and reverse primers are 0.5 μL. The PCR procedure was as follows: Pre-denaturation at 94°C for 5 mins, Denatured at 94°C for 30 s, annealed at 50-64°C for 30 s, extended at 72°C for 30 s, 27 cycles, extended at 72°C for 7 mins. The PCR amplification products were detected by 8% polyacrylamide gel electrophoresis.

#### 2.2.3 Assay of leaf and thorn traits

Leaf traits were determined according to the methods described by Qiu et al. (2021). In mid-September 2023, mature or nearly mature leaves of jujube bearing shoots were collected from the trees in the morning from 8:00 to 9:00. The leaves were placed in a zip-lock bag and stored in an ice box to bring back to the laboratory. The leaves were washed with distilled water and placed on filter paper to remove the water. Then, the leaves were scanned by Wanshen LA-S series plant image analyzer. The leaf length, leaf width, leaf area and leaf circumference were measured by ImageJ-64, and the leaf shape index (leaf length/leaf width) was calculated. Each test material has 30 replications, and the average value was calculated.

Thorn traits were assessed using the method proposed by Pu (1990) with some modifications. In March 2023, the middle of the secondary branch thorns were selected and the digital display vernier caliper (0.01 mm) was used to measure the straight thorn length, straight thorn thickness, hooked thorn length and thickness. Each test material has 6 replications and the average value was calculated.

#### 2.2.4 Assay of stomatal traits

Stomatal mounts were made by the nail polish smearing and tearing method (Miller-Rushing et al., 2009). In mid-September 2023, the leaves of jujube bearing shoots on different directions around the canopy were randomly picked. Thin layer of nail polish, approximately 1.5cm wide and 2cm long, was applied to the middle of the underside of the leaves. After allowing it to settle down for 3~6 min, the nail polish was carefully removed with the help of tweezers and placed it on the slide. 1~2 drops of water were added and on the top and cover glass was placed on its top to prepare a temporary slide. The temporary slide was observed with a 20×objective lens of an electron microscope (BX51 Olympus). Each test material has 6 replications, each time 2~4 view fields were randomly selected, a total of 15~20 view fields. The length and width of 50 stomata were measured with ImageJ-64, and the density of stomata was calculated.

Calculation formula of stomatal density (number·mm^−2^) = number of stomata in the field of view/field area.

#### 2.2.5 Assay of fruit size and fruit kernel traits

Fruit size and kernel traits were determined according to the method proposed by Liu et al. (2013). In September-October 2022-2023, 30 semi-red fruits with the same size and without any disease were picked from the middle of the jujube bearing shoots in different directions of canopy. The fruit weight, fruit longitudinal and transverse diameters were measured by electronic balance (0.01 g) and digital vernier caliper (0.01 mm), and the fruit shape index (fruit length/fruit diameter) was calculated. Each test material has 30 replications, and the average value was taken.


$$\begin{array}{c} \text{Edible rate}={\text{(W}}_{\text{F}}-{\text{W}}_{\text{C}})/\text{WF}\\ \times 100\%\left({\text{W}}_{\text{F}}\;{\text{is fruit weight, W}}_{\text{C}}\;\text{is single fruit kernel weight}\right) \end{array}$$

#### 2.2.6 Assay of fruit outer quality and sensory traits

The semi-red fruits with the same size and without any disease were picked from the middle of the jujube bearing shoot in different directions of canopy. According to the classification criteria and professional terms of ‘Chinese Jujube Germplasm Resources’ (Liu and Wang, 2009), the classification, description and recording were carried out. Two fruit outer quality traits (fruit shape and color) and four sensory quality traits (peel thickness, flesh color, flesh texture and fruit flavor) were determined by eyeballing method and group tasting method. Each test material has 10 replications and proportion of each trait was calculated.

#### 2.2.7 Assay of fruit nutritional traits

Use a laboratory beater to mash and mix 30 jujube pulps, repeat 3 times for each test material and calculate the average value. Soluble solids content: Automatic benchtop refractometer (RX-5000α, ATAGO, Japan) (Mao et al., 2008) was used for determination of soluble solids content. Vitamin C content: The molybdenum blue colorimetric method (Chen et al., 2021) was used to determine the vitamin C content. Soluble sugar content: The anthrone sulfuric acid colorimetric method (Li et al., 2021) was used to determine the soluble sugar content. Titratable acid content: The acid-base neutralization titration method (Wang et al., 2021) was used to determine the titratable acid content.

### 2.3 Date processing

Excel 2016 was used to sort out the data, and SPSS 26.0 was used to analyze the data. Genetic variation formula:


CV=SD/F×100%

MP=(P1+P2)/2

MPH=(F-MP))/MP)×100%

RH=(NH/N)×100%

RL=(NL/N)×100%

In the formula, CV is the variation coefficient, SD is the standard deviation of each trait. MP is the mid-parent value, P1 is the mean value of ‘Dongzao’ traits, P2 is the mean value of ‘Chenguang’ traits. MPH is the mid-parent heterosis, F is the mean values of progeny traits. RH is ultra-high parent ratio, NH is higher than the number of high parent progeny, RL is ultra-low parent ratio, NL is lower than the number of low parent progeny, N is the total number of hybrid progeny.

## 3 Results

### 3.1 Hybrid progeny identification

#### 3.1.1 Ploidy identification of hybrid progeny

**Figure 1:**
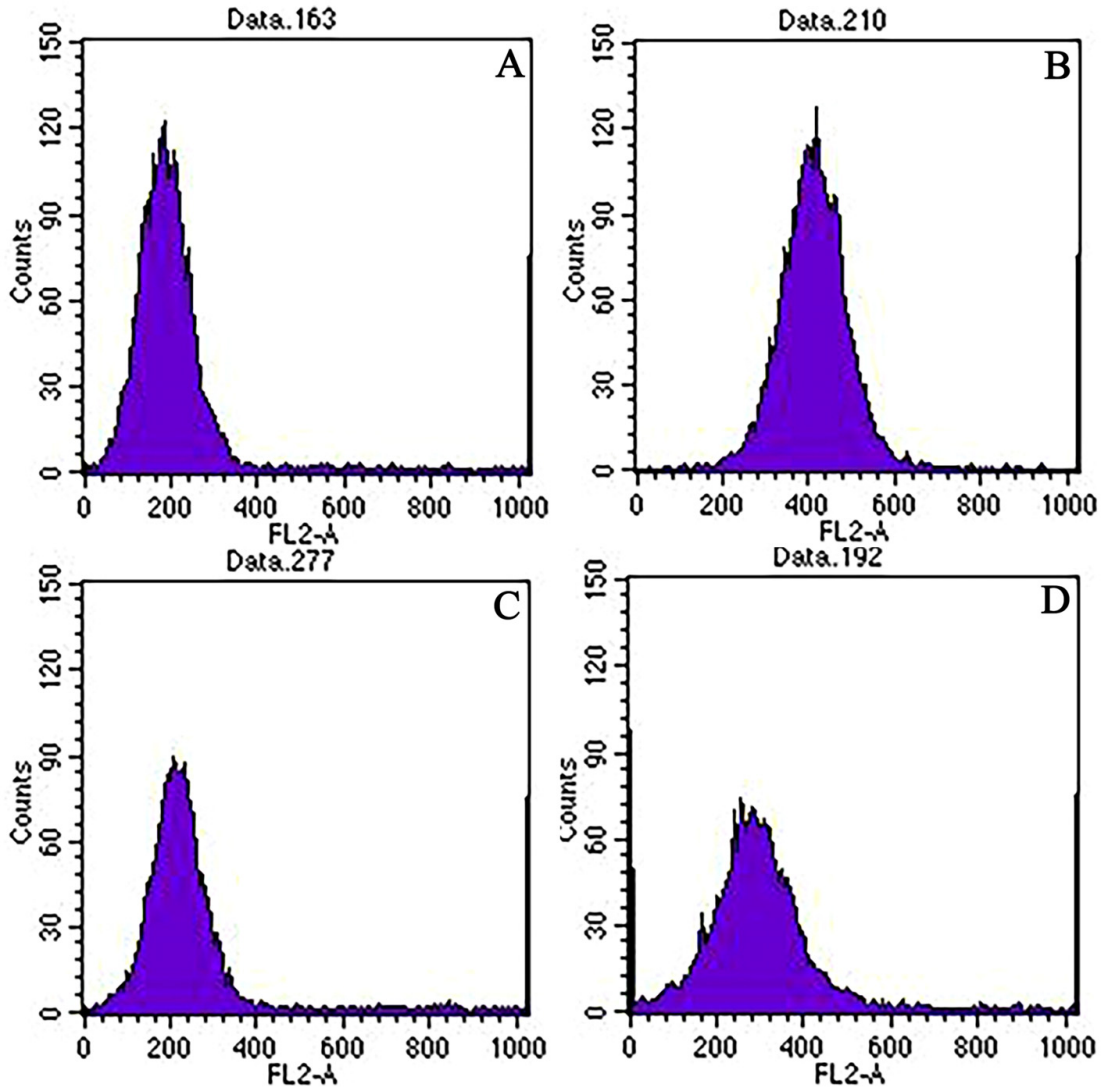
Ploidy detection of the two parents and hybrid progeny by flow cytometry. Note: **(A)**: 'Dongzao'; **(B)**: 'Chenguang '; **(C)**: Triploid progeny; **(D)**: Diploid progeny; The abscissa axis represented the fluorescence intensity value, the ordinate axis represented the number of cell nucleus, and the location of the peak represented the ploidy of progeny..

The ploidy of the two parents (‘Dongzao’×’Chenguang’) and hybrid progeny was detected by flow cytometry. The flow cytometry of the two parents showed clear and stable peaks. Each had a high single peak (**Figures 1A, B**). Because the female parent ‘Dongzao’ is diploid, the peak of chromosome fluorescence intensity appeared at about 200, the male parent ‘Chenguang’ is tetraploid, the peak of chromosome fluorescence intensity appeared at about 400. In addition, the peak of chromosome fluorescence intensity of progeny appeared at about 300, which demonstrated they were triploid progeny (**Figure 1C**), while the peak appeared at about 200, which demonstrated they were diploid progeny (**Figure 1D**). The statistical results of ploidy detection in the progeny were as follows: out of 124 hybrid progeny, 68 triploids were detected, accounting for 54.84%, and 56 diploids were detected, accounting for 45.16%.

#### 3.1.2 SSR identification of the progeny

**Figure 2:**
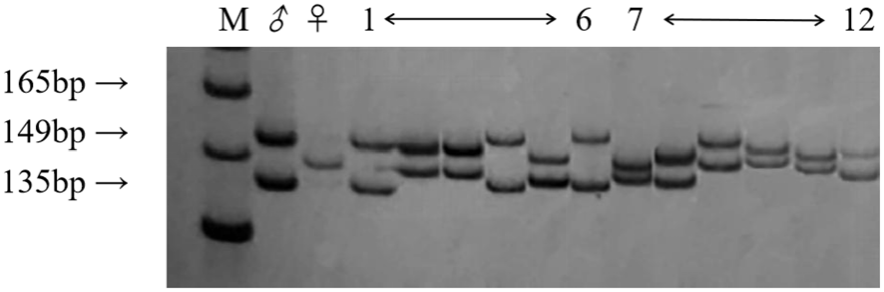
The band amplification of primer JSSR239 in parents and some diploid and triploid progeny. (M): DNA maker; (♂): The female parent; (♀): The male parent; (1~6): Triploid progeny; (7~12): Diploid progeny.

To conform whether the hybrid progeny are true hybrids, 5 SSR primers were used to identify the diploid and triploid progeny obtained from the hybridization of ‘Dongzao’×’Chenguang’ (**Figure 2**). Having paternal specific bands or both parents specific bands is considered to be the key evidence for the identification of true hybrids. The results showed that 118 of the 124 hybrids were identified as true hybrids with specific bands of paternity or both parents, including 50 diploid hybrids and 68 triploid hybrids. By comparing the bands of 5 SSR primers (**Table 1**), Primer JSSR239 had the best efficiency in the identification of triploid progeny. The hybrid rate of triploid progeny identified by primer JSSR239 was the highest. Among them, 38 strains had both parent specific bands and 12 strains had paternal specific bands. The hybrid rate of diploid progeny identified by primer JSSR131 was the highest. Among them, 25 strains had both parent specific bands and 4 strains had paternal specific bands.

**Table 1:** Distribution of different SSR loci in diploid and triploid progeny.

Primers	Ploidy	Markers presented in both parents (plants/percentage%)	Markers presented only in female parent (plants/percentage%)	Markers presented only in male parent (plants/percentage%)	New Markers presented in progeny (plants/percentage%)	Markers absented in progeny (plants/percentage%)
*JSSR131*	3x	40/58.82	22/32.35	6/8.83	0/0.00	0/0.00
	2x	25/44.64	27/48.21	4/7.14	0/0.00	0/0.00
*JSSR214*	3x	0/0.00	37/54.41	30/44.12	0/0.00	1/1.47
	2x	1/1.79	50/89.28	3/5.36	0/0.00	2/3.57
*JSSR239*	3x	38/55.88	16/23.52	12/17.65	0/0.00	2/2.94
	2x	8/14.29	27/48.21	18/32.14	0/0.00	3/5.36
*JSSR314*	3x	0/0.00	36/52.94	21/30.88	4/5.88	7/10.29
	2x	0/0.00	17/30.36	9/16.07	29/51.79	1/1.79
*JSSR318*	3x	0/0.00	20/29.41	47/69.12	0/0.00	1/1.47
	2x	0/0.00	53/94.64	3/5.36	0/0.00	0/0.00

### 3.2 Analysis of leaf traits in triploid progeny

#### 3.2.1 Comparative analysis of leaf traits

**Figure 3:**
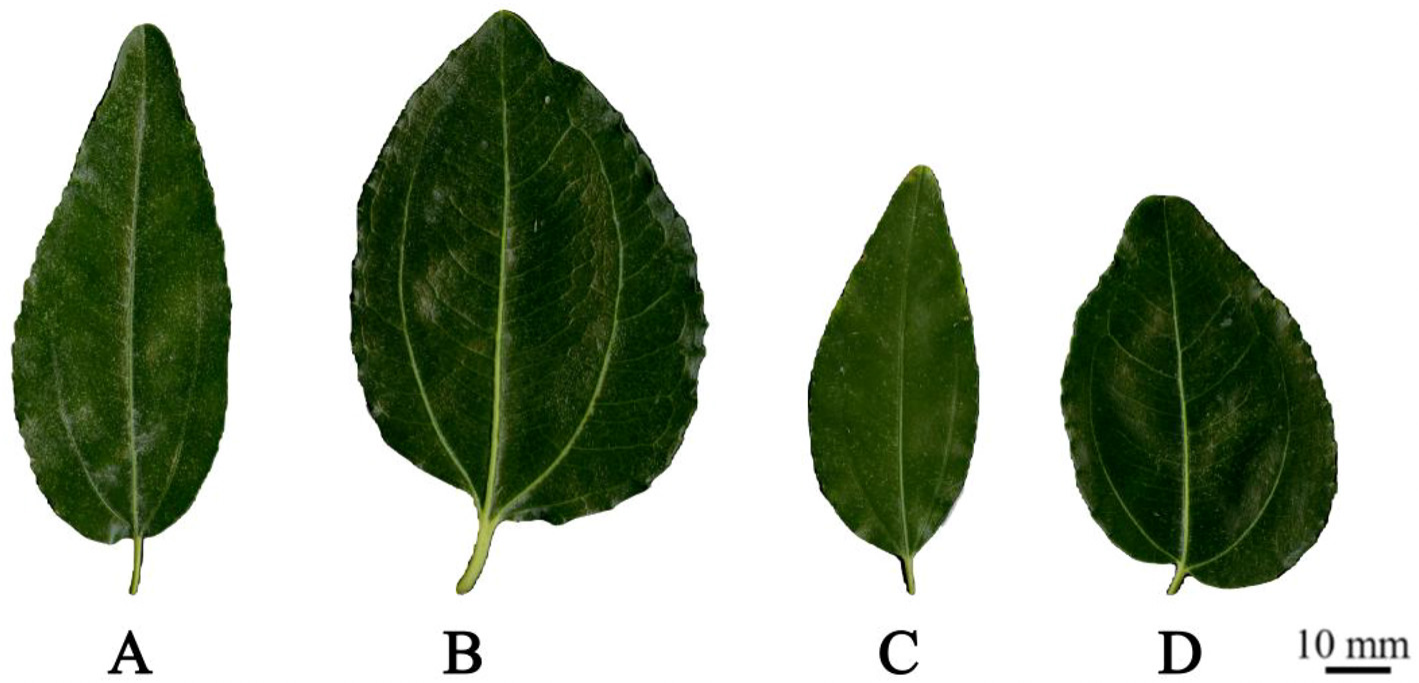
Leaf morphology of two parents, diploid and triploid progeny. **(A)** Leaf of ‘Dongzao’; **(B)** Leaf of ‘Chenguang’; **(C)** Leaf of diploid progeny; **(D)** Leaf of triploid progeny.

By comparing the leaf size of triploid progeny with that of parents and diploid progeny, it was found that the leaf size of the triploid progeny was significantly larger than that of the diploids (**Figure 3**). The leaf length, width, area and circumference of triploid and diploid progeny were lower than those of both parents, and the leaf shape index was between that of both parents. The leaf shape index of triploid progeny was significantly lower than that of diploids, and the leaf width and area was significantly higher than those of diploids (**Table 2**), showing the characteristics of round leaves. With the increase of chromosome ploidy, the leaves of triploid progeny were shorter and wider, and the leaf shape index was smaller, which was significantly different from that of diploid progeny. This feature can be used as a morphological indexes to preliminarily determination of the ploidy of jujube hybrid progeny.

**Table 2:** Comparison of leaf traits between triploid and diploid progeny.

Leaf traits	Mean ± SD				
	Leaf length/mm	Leaf width/mm	Leaf area/mm^2^	Leaf circumference/mm	Leaf shape index
‘Dongzao’ (2x)	78.92 ± 5.69	33.44 ± 2.45	1863.16 ± 231.71	210.52 ± 14.91	2.36 ± 0.13
‘Chenguang’ (4x)	66.19 ± 8.35	49.16 ± 6.86	2394.98 ± 607.24	213.98 ± 27.02	1.35 ± 0.11
Triploid progeny (3x)	48.16 ± 7.98	30.41 ± 5.44**	1095.45 ± 365.36**	147.23 ± 26.18	1.60 ± 0.21**
Diploid progeny (2x)	49.11 ± 7.59	23.58 ± 4.21	849.64 ± 271.02	137.18 ± 21.78	2.11 ± 0.24

t-test of independence between triploid and diploid progeny, * represented the significant difference at *p*<0.05 level, and ** represented the significant difference at *p*<0.01 level.

**Figure 4:**
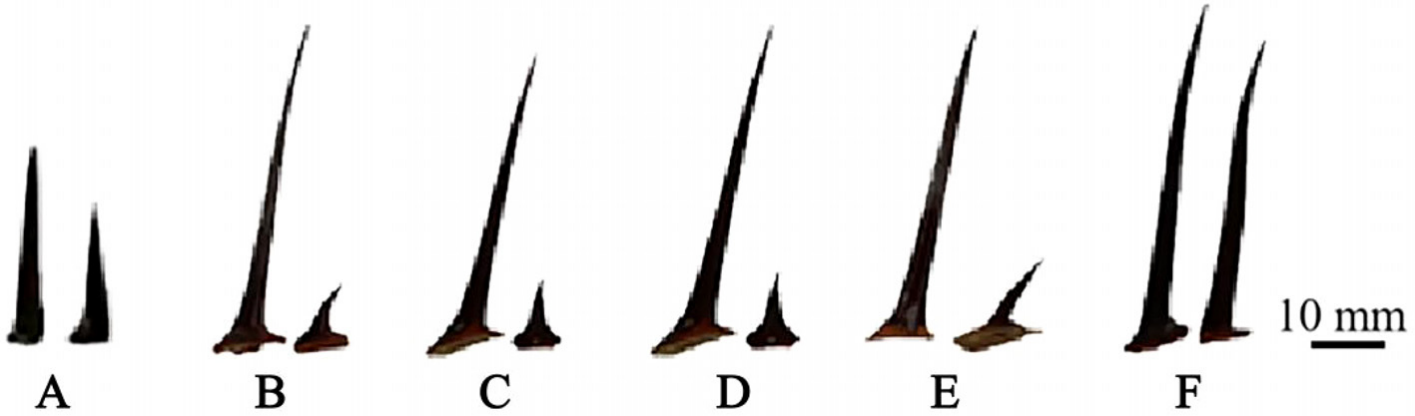
Thorn morphology comparison of two parents and diploid and triploid progeny. **(A)** Thorn of ‘Chenguang’. **(B, C)** Thorn of diploid progeny. **(D, E)** Thorn of triploid progeny. **(F)**: Equal thorns (triploid hybrid T92).

By comparing the thorn size of triploid progeny with that of parents and diploid progeny, it was found that the thorn size of triploid progeny was significantly larger than the diploid (**Figure 4**). The female parent ‘Dongzao’ showed thorn degradation, while straight and hook thorn of the male parent ‘Chenguang’ are pairs of thorns. In the diploid and triploid progeny, there was no thorn degradation, but only one triploid hybrid presents equal thorns between straight and hooked thorn (**Figure 4F**). The thorn traits of triploid progeny were greater than that of the male parent ‘Chenguang’. The straight thorn length, hooked thorn length in triploid progeny was significantly higher than that of diploid, showing the characteristics of larger thorn (**Table 3**). The straight thorn thickness of triploid progeny was lower than that of diploid progeny. It can be seen that the thorn of triploid progeny was longer and thicker, which was different from that of diploid progeny. This feature can be used as an auxiliary index to judge the ploidy of jujube hybrid progeny.

**Table 3:** Comparison of thorn traits between triploid and diploid progeny.

Thorn traits	Mean ± SD			
	Straight thorn length/mm	Straight thorn thickness/mm	Hooked thorn length/mm	Hooked thorn thickness/mm
‘Dongzao’ (2x)	0.00	0.00	0.00	0.00
‘Chenguang’ (4x)	4.65 ± 1.11	1.09 ± 0.34	4.42 ± 1.45	0.89 ± 0.24
Triploid progeny (3x)	21.58 ± 5.78**	2.24 ± 0.28	5.60 ± 1.92**	1.18 ± 0.20
Diploid progeny (2x)	15.09 ± 5.19	2.34 ± 0.34	3.83 ± 1.57	1.15 ± 0.19

t-test of independence between triploid and diploid progeny, * represented the significant difference at *p*<0.05 level, and ** represented the significant difference at *p*<0.01 level.

**Figure 5:**
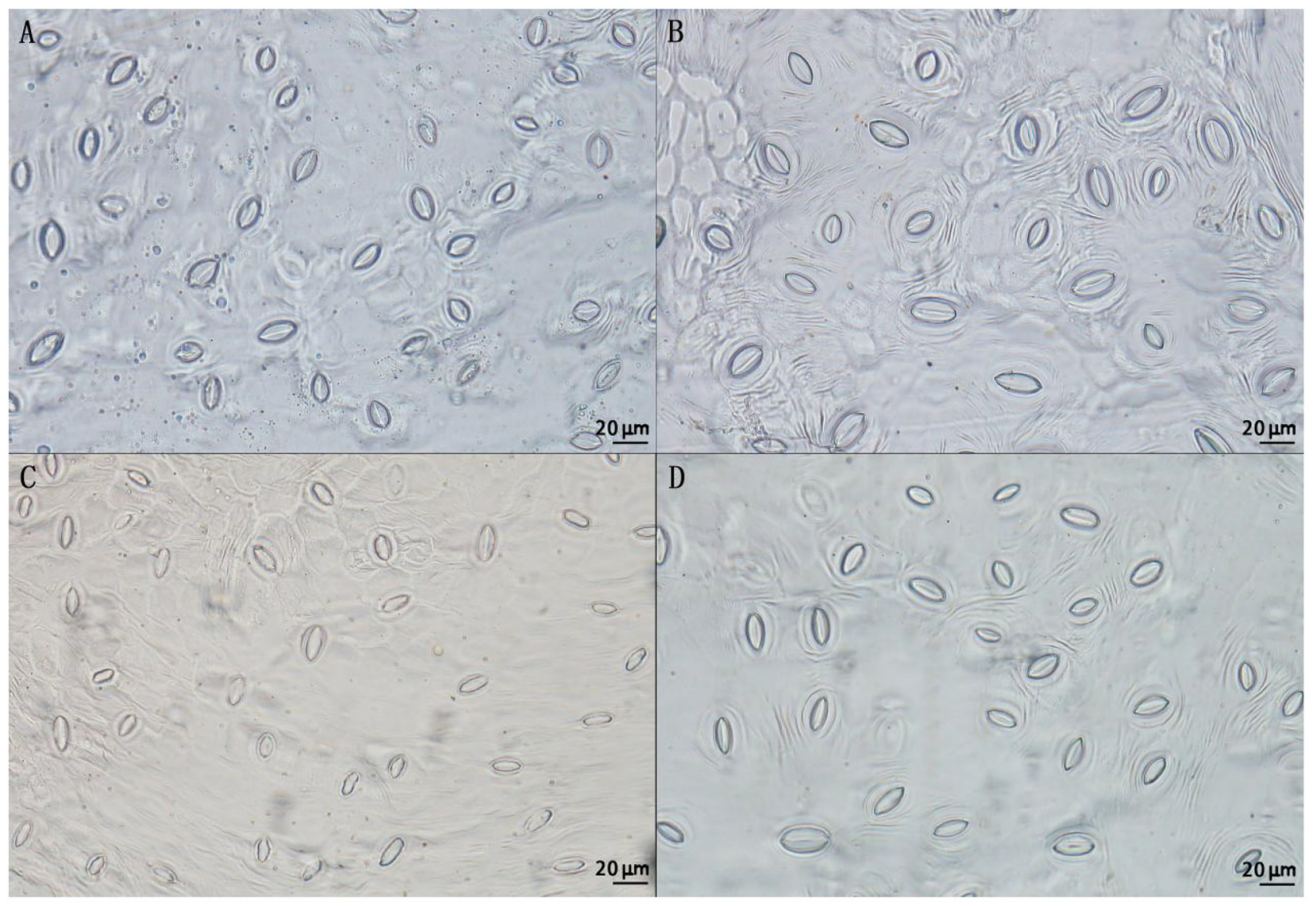
Stomatal morphology of two parents and diploid and triploid progeny. **(A)** Stomata of ‘Dongzao’; **(B)** Stomata of ‘Chenguang’; **(C)** Stomata of diploid progeny; **(D)** Stomata of triploid progeny.

By comparing the stomatal size of triploid progeny with that of parents and diploid progeny, it can be seen that the stomatal size of triploid progeny was significantly larger than the diploid (**Figure 5**). The stomatal length, stomatal density and stomatal width of triploid offsprings were intermediate between the parent values. The stomatal length and stomatal width of triploid progeny were significantly higher than those of diploid, but the stomatal density of triploid progeny was significantly lower than that of diploid (**Table 4**), showing the characteristics of larger stomata. Thus, with the increase of chromosome ploidy, the stomata of triploid progeny were larger and rounder, while the stomatal density decreased, indicating significant difference from the stomatal size traits of diploid progeny. This characteristic can also be used as one of the morphological indicators to identify whether the plant chromosome had been doubled.

**Table 4:** Comparison of stomatal traits between triploid and diploid progeny.

Stomatal traits	Mean ± SD		
	Stomatal length/μm	Stomatal width/μm	Stomatal density/(number·μm^2^)
‘Dongzao’ (2x)	16.43 ± 1.88	8.48 ± 1.41	316.09 ± 80.20
‘Chenguang’ (4x)	24.98 ± 2.57	10.48 ± 1.53	181.88 ± 34.04
Triploid progeny (3x)	20.99 ± 2.51**	9.25 ± 1.31**	214.59 ± 32.35**
Diploid progeny (2x)	18.72 ± 1.82	8.28 ± 0.90	256.69 ± 27.58

t-test of independence between triploid and diploid progeny, * represented the significant difference at *p*<0.05 level, and ** represented the significant difference at *p*<0.01 level.

#### 3.2.2 Variation analysis of leaf traits in triploid progeny

The variation in leaf traits of triploid progeny was conducted statistical analysis (**Table 5**). The variation coefficients of leaf indicators in triploid progeny ranged from 13.13% to 33.35%, indicating that leaf indicators were widely separated in triploid progeny. The mid-parent heterosis of leaf indicators in triploid progeny were negative, and the ultra-low parent ratio in leaf indicators was more than 70%, showing an obvious tendency of low genetic variation. The variation coefficient of thorn indicators in triploid progeny ranged from 12.50~34.29%, indicating that the thorn indicators were separated to a greater degree in triploid progeny. The mid-parent heterosis of thorn indicators in triploid progeny was positive, and the ultra-high parent ratio of thorn indicators was more than 70%, showing a trend of high genetic variation. The variation coefficient of stomatal indicators in triploid progeny ranged from 11.96~15.08%, indicating that stomatal indicators were widely separated in triploid progeny. The mid-parent heterosis of stomatal width and density in triploid progeny were negative, and the super-high parent ratio in stomatal width and density was small, indicating a trend from intermediate to lower genetic variation. The mid-parent heterosis of stomatal length in triploid progeny was 1.35%, and the ultra-low parent ratio was 3.28% in stomatal length, showing a trend of intermediate genetic variation.

**Table 5:** Variation analysis of leaf traits in triploid progeny.

Leaf traits		Variation coefficient/%	Variation range	Mid-parent heterosis/%	Ultra-high parent ratio/%	Ultra-low parent ratio/%
Leaf traits	Leaf length/mm	16.57	34.18~70.33	-33.63	0	98.33
	Leaf width/mm	17.89	17.63~46.06	-26.37	0	71.67
	Leaf area/mm^2^	33.35	485.52~2363.52	-48.55	0	96.67
	Leaf circumference/mm	17.78	101.36~227.05	-30.63	1.67	96.67
	Leaf shape index	13.13	1.36~2.43	-13.98	1.67	0
Thorn traits	Straight spine length/mm	26.78	8.92~36.06	826.18	100.00	–
	Straight barbed wire thickness/mm	12.50	1.54~2.85	305.45	100.00	–
	Hooked barbs length/mm	34.29	2.27~14.15	153.39	74.58	–
	Barbed wire thickness/mm	16.95	0.87~1.60	162.22	93.22	–
Stomatal traits	Stomatal length/μm	11.96	15.05~26.53	1.35	6.56	3.28
	Stomatal width/μm	14.16	7.21~12.64	-2.43	14.75	27.87
	Stomatal density /(number·μm^2^)	15.08	114.77~271.56	-13.82	0.00	16.39

### 3.3 Analysis of fruit traits in triploid progeny

#### 3.3.1 Comparative analysis of fruit outer and sensory quality

The distribution of fruit outer and sensory quality of triploid progeny were shown in **Table 6**. From the perspective of fruit shape, the fruit outer traits of diploid and triploid progeny were quite different. The fruit shape of triploid progeny was predominantly oblate, accounting for 42.00% and 43.14% respectively in two consecutive years. However, the proportion of oval and round shapes was the smallest. There were 5 and 6 malformed fruits in the two years. The fruit color of the hybrid progeny showed separation of four traits, among which the diploid and triploid progeny were mostly red, accounting for more than 85.00%, and it indicated the inheritance of fruit color traits was relatively stable. It was one of the breeding objectives to select plants with thin pericarp. By comparing the peel thickness of diploid and triploid progeny, it was found that there were 27 and 11 plants with thin peel among triploid progeny during the two years, accounting for 54.00% and 21.57% of triploid progeny, respectively. It demonstrates a significant thinning trend of peel thickness in triploid progeny. From the perspective of flesh color, the proportion of light green flesh in triploid progeny was more than 80.00%, which was similar as compared to parents. Flesh texture (Wang et al., 2016) and fruit flavor (Wang et al., 2016) are important factors affecting fruit quality. From the perspective of flesh texture, both parents had crisp taste. There were 17 and 14 plants with crisp flesh in triploid progeny in 2 years, accounting for 34.00% and 27.45% of triploid progeny, respectively. These findings indicated that the flesh texture of triploid progeny could be inherited stably. From the perspective of fruit flavor, the parents were mainly sweet-sour, but the sour-sweet flavors of diploid and triploid progeny accounted for a large proportion, which the sour-sweet flavors of triploid progeny accounted for 46.00% and 45.10% respectively in two years.

**Table 6:** Distribution of fruit outer and sensory traits in diploid and triploid progeny.

Fruits	Years	Parents		Number of progeny (plants/percentage%)									
		‘Dongzao’ (2x)	‘Chenguang’ (4x)	Ploidy	Distribution of fruit outer and sensory traits								
Fruit shape					Oblate	Flat cylinder	Obovate	Ovoid		Globose	Cylinder	Oblong globose	Abnormality
	2022	Oblate	Obovate	2x	0 (0.00)	0 (0.00)	1 (12.50)	1 (12.50)		1 (12.50)	2 (25.00)	3 (37.50)	0 (0.00)
				3x	21 (42.00)	4 (8.00)	4 (8.00)	0 (0.00)		3 (6.00)	7 (14.00)	6 (12.00)	5 (10.00)
	2023	Oblate	Obovate	2x	2 (14.29)	0 (0.00)	1 (7.13)	0 (0.00)		2 (14.29)	2 (14.29)	7 (50.00)	0 (0.00)
				3x	22 (43.14)	6 (11.77)	6 (11.77)	2 (3.92)		1 (1.96)	3 (5.88)	5 (9.80)	6 (11.76)
Fruit color					Light red		Red			Mauve		Reddish brown	
	2022	Red	Red	2x	0 (0.00)		7 (87.50)			0 (0.00)		1 (12.50)	
				3x	1 (2.00)		43 (86.00)			6 (12.00)		0 (0.00)	
	2023	Red	Red	2x	0 (0.00)		13 (92.86)			1 (7.14)		0 (0.00)	
				3x	2 (3.92)		46 (90.2)			2 (3.92)		1 (1.96)	
Peel thickness					Thin		Intermediate			Thick			
	2022	Thin	Thin	2x	6 (75.00)		2 (25.00)			0 (0.00)			
				3x	27 (54.00)		22 (44.00)			1 (2.00)			
	2023	Thin	Thick	2x	5 (35.71)		5 (35.71)			4 (28.58)			
				3x	11 (21.57)		24 (47.06)			16 (31.37)			
Flesh color					White		Light green		Green				
	2022	Light green	Light green	2x	4 (50.00)		4 (50.00)		0 (0.00)				
				3x	2 (4.00)		46 (92.00)		2 (4.00)				
	2023	Light green	White	2x	4 (28.57)		9 (64.29)		1 (7.14)				
				3x	4 (7.84)		43 (84.32)		4 (7.84)				
Flesh texture					Loose		Crisp		Intermediate			Compact	
	2022	Crisp	Crisp	2x	2 (25.00)		1 (12.50)		5 (62.50)			0 (0.00)	
				3x	10 (20.00)		17 (34.00)		19 (38.00)			4 (8.00)	
	2023	Crisp	Crisp	2x	2 (14.29)		7 (50.00)		2 (14.29)			3 (21.42)	
				3x	1 (1.96)		14 (27.45)		25 (49.02)			11 (21.57)	
Fruit flavor					Sour		Sweet-sour		Sour-sweet			sweet	
	2022	Sweet-sour	Sweet-sour	2x	6 (75.00)		0 (0.00)		1 (12.50)			1 (12.50)	
				3x	1 (2.00)		5 (10.00)		23 (46.00)			21 (42.00)	
	2023	Sweet-sour	Sour-sweet	2x	4 (28.57)		1 (7.14)		6 (42.86)			3 (21.43)	
				3x	3 (5.88)		16 (31.37)		23 (45.10)			9 (17.65)	

#### 3.3.2 Comparative analysis of fruit and fruit kernel traits

**Figure 6:**
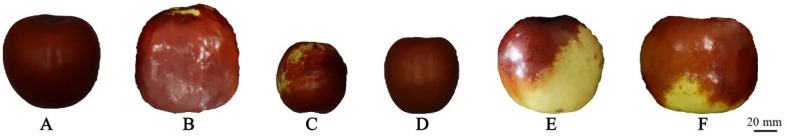
Fruit morphology of two parents and diploid and triploid progeny. **(A)** Fruit of ‘Dongzao’; **(B)** Fruit of ‘Chenguang’; **(C, D)** Fruits of diploid progeny; **(E, F)** Fruits of triploid progeny.

By comparing the fruit size of triploid progeny with that of parents and diploid progeny, it can be seen that the fruit size of the triploid progeny was significantly larger than the diploid (**Figure 6**). The single fruit weight, fruit diameter and fruit length of triploid progeny were larger than that of female parent ‘Dongzao’, and smaller than that of male parent ‘Chenguang’, but the fruit shape index was larger than that of parents. The single fruit weight, fruit diameter, fruit length and fruit shape index of triploid progeny were significantly higher than those of diploid. The edible rate of triploid progeny was higher and significantly higher than that of diploid (**Table 7**), showing the characteristics of large fruit type. It can be seen that after doubling, the single fruit weight, fruit diameter, fruit length, fruit shape index and edible rate all increased, which was different from the fruit size traits of diploid progeny, laying a foundation for the breeding of excellent large fruit progeny.

**Table 7:** Comparison of fruit size traits between triploid and diploid progeny.

Fruit size traits	Years	Mean ± SD				
		Single fruit weight/g	Fruit length/mm	Fruit diameter/mm	Fruit shape index	Edible rate/%
‘Dongzao’ (2x)	2022	10.39 ± 2.03b	28.00 ± 2.10c	26.84 ± 1.99b	0.96 ± 0.03a	94.37 ± 1.68b
	2023	8.76 ± 2.24c	32.96 ± 2.92b	22.94 ± 1.81c	0.70 ± 0.09d	96.67 ± 1.14b
‘Chenguang’ (4x)	2022	36.43 ± 4.88a	45.06 ± 2.57a	39.48 ± 3.16a	0.88 ± 0.07b	97.32 ± 0.49a
	2023	33.58 ± 9.07a	41.87 ± 4.56a	39.89 ± 3.69a	0.96 ± 0.07b	98.88 ± 0.72a
Triploid progeny (3x)	2022	29.56 ± 14.82a	39.09 ± 8.53b	37.27 ± 6.44a	0.97 ± 0.10a	97.58 ± 0.98a
	2023	24.56 ± 11.37b	33.57 ± 7.93b	34.65 ± 6.22b	1.05 ± 0.13a	97.35 ± 1.36b
Diploid progeny (2x)	2022	9.65 ± 8.91b	28.15 ± 9.66c	23.34 ± 7.30b	0.84 ± 0.07b	94.77 ± 2.47b
	2023	7.37 ± 8.61c	22.49 ± 7.97c	20.06 ± 8.22d	0.88 ± 0.18c	92.52 ± 3.50c

Triploid and diploid progeny of the same year were analyzed for significance analysis, and different lowercase letters marked in the table indicated significant difference at *p*< 0.05 level.

**Figure 7:**
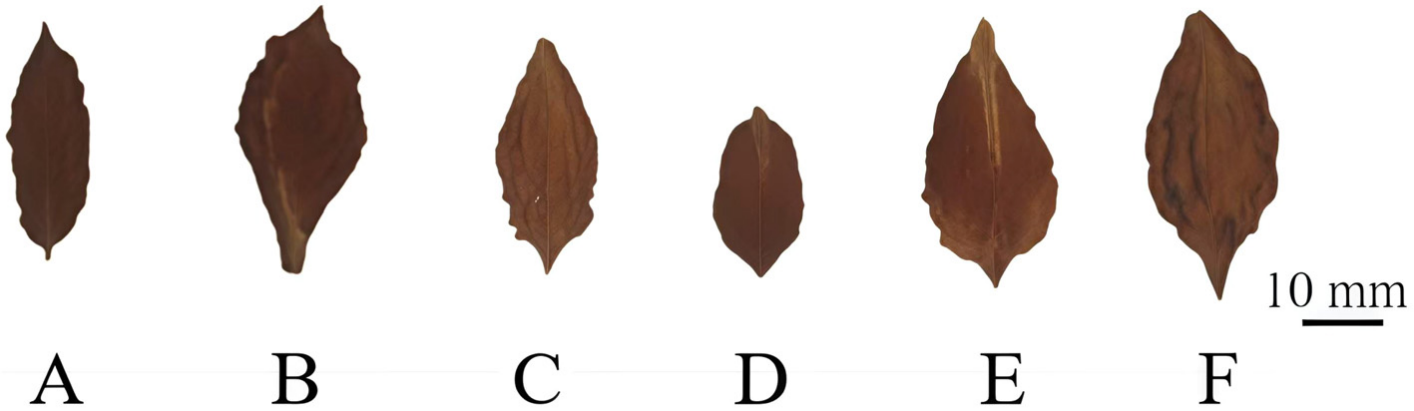
Kernel morphology of two parents and diploid and triploid progeny. **(A)** Kernel of ‘Dongzao’; **(B)** Kernal of ‘Chenguang’; **(C, D)** Kernal of diploid progeny; **(E, F)** Kernal of triploid progeny.

By comparing the size of fruit kernel of triploid progeny with that of parents and diploid progeny, it can be seen that the size fruit kernel of the triploid progeny was significantly larger than the diploid (**Figure 7**). The single fruit kernel weight, longitudinal diameter and transverse diameter of triploid progeny were larger, but fruit kernel index was smaller when compared with female parent ‘Dongzao’. The single fruit kernel weight, transverse diameter, longitudinal diameter of triploid offsprings were significantly higher and the fruit kernel index was significantly lower as compared to diploid progeny (**Table 8**), and it demonstrated the characteristics of larger fruit kernel. Our results demonstrated that with the increase of chromosome ploidy, single fruit kernel weight, longitudinal diameter and transverse diameter also increased, but decrease in the index of fruit kernel observed, which was significantly different from the size of fruit kernel in diploid progeny but consistent with the trends in fruit size of triploid progeny. These findings indicated that this characteristic can be used as a key indicator to determine whether the hybrid progeny have undergone chromosomal doubling.

**Table 8:** Comparison of kernel traits between triploid and diploid progeny.

Kernel size traits	Years	Mean ± SD			
		Single fruit kernel weight/g	Kernel longitudinal diameter/mm	Kenel transverse diameter/mm	Kernel index
‘Dongzao’ (2x)	2022	0.56 ± 0.11b	19.49 ± 2.20b	7.84 ± 0.79c	2.50 ± 0.31a
	2023	0.27 ± 0.04b	15.66 ± 1.21c	6.36 ± 0.34c	2.46 ± 0.15a
‘Chenguang’ (4x)	2022	0.96 ± 0.16a	22.56 ± 0.68a	10.27 ± 0.48a	2.20 ± 0.13b
	2023	0.36 ± 0.20b	20.55 ± 2.79a	8.63 ± 1.57a	2.46 ± 0.55a
Triploidprogeny (3x)	2022	0.62 ± 0.31b	20.52 ± 4.66ab	9.34 ± 1.39b	2.23 ± 0.37b
	2023	0.55 ± 0.29a	18.05 ± 4.38b	9.28 ± 1.56a	1.97 ± 0.37b
Diploid progeny (2x)	2022	0.36 ± 0.20c	16.66 ± 4.59c	7.12 ± 1.50c	2.38 ± 0.55ab
	2023	0.33 ± 0.20b	14.80 ± 4.37c	7.32 ± 1.65b	2.07 ± 0.42b

Triploid and diploid progeny of the same year were analyzed for significance analysis, and different lowercase letters marked in the table indicated significant difference at *p*< 0.05 level.

#### 3.3.3 Comparative analysis of fruit nutritional traits

By comparing the fruit nutritional traits of triploid progeny with that of parents and diploid progeny, it was concluded that the soluble solids and soluble sugar content of triploid progeny were between those of parents, while the vitamin C content of triploid progeny was less than that of diploid. The titratable acid content of triploid progeny was significantly lower than that of diploid, and the soluble solids and soluble sugar content were higher than those of diploid (**Table 9**), showing the characteristics of high sugar and low acid. Thus, we proposed that after doubling the triploid offsprings was higher than the diploid in terms of sugar substances, but less than the diploid in terms of acid substances. Therefore, it was speculated that the phenomenon of high sugar and low acid was an important feature of triploid offsprings.

**Table 9:** Comparison of fruit nutritional traits between triploid and diploid progeny.

Fruit nutritional traits	Years	Mean ± SD			
		Vitamin C content/(mg/100g)	Soluble solid content/%	Soluble sugar content/%	Titratable acid content/%
‘Dongzao’ (2x)	2022	289.23 ± 20.18	42.13 ± 0.12	36.80 ± 1.87	0.60 ± 0.18
	2023	253.96 ± 4.58	41.48 ± 1.72	24.72 ± 1.34	0.49 ± 0.04
‘Chenguang’ (4x)	2022	301.19 ± 15.48	33.00 ± 0.20	23.47 ± 4.75	0.54 ± 0.18
	2023	372.73 ± 19.50	32.96 ± 0.23	22.08 ± 0.73	0.56 ± 0.03
Triploid progeny (3x)	2022	268.44 ± 38.08*	32.92 ± 3.72	29.19 ± 6.46	0.50 ± 0.09**
	2023	429.25 ± 88.28	37.53 ± 3.94*	24.00 ± 2.35**	0.61 ± 0.10**
Diploid progeny (2x)	2022	297.82 ± 50.59	30.70 ± 3.60	26.10 ± 4.06	0.70 ± 0.17
	2023	467.81 ± 143.95	34.23 ± 4.88	20.35 ± 2.90	0.89 ± 0.23

t-test of independence between triploid and diploid progeny, * represented the significant difference at *p*<0.05 level.

#### 3.3.4 Variation analysis of fruit traits in triploid progeny

The variation of fruit size traits in triploid progeny was analyzed (**Table 10**). The variation coefficient of fruit size indicators in triploid progeny ranged from 1.00~50.14%, indicating that fruit size indicators were widely separated in triploid progeny. The mid-parent heterosis of single fruit weight, fruit length and fruit shape index in triploid progeny was positive with a high super parent ratio. It indicated a trend of high genetic variation. The edible rate of triploid progeny showed a trend of intermediate genetic variation. The ultra-low parent heterosis ratio of fruit size indicators in triploid progeny was positive, and the ultra-low parent heterosis appeared. The variation coefficient of fruit kernel indicators in triploid progeny ranged from 14.88~52.73%, indicating that there was a wide separation in fruit kernel size indicators in triploid progeny. The mid-parent heterosis of fruit kernel longitudinal diameter and fruit kernel index in triploid progeny was negative, and the ultra-low parent ratio was high, indicating a trend of low genetic variation. The mid-parent heterosis of the transverse diameter of the fruit kernel in triploid progeny was positive, and the super-high parent ratio was high, demonstrating a trend of large genetic variation. The variation coefficient of fruit nutritional indicators in triploid progeny ranged from 9.79~22.13% indicating that fruit nutritional indicators were widely separated in triploid progeny. Due to different years, the variation of fruit nutritional indicators has a certain degree of differences. In 2022, the mid-parent heterosis of fruit nutritional indicators in triploid progeny were negative, and the ultra-low parent ratio was higher, showing a trend of low genetic variation. In 2023, the mid-parent heterosis ratio of fruit nutritional indicators in triploid progeny were positive, the ultra-high parent of vitamin C content was higher, showing a trend of high genetic variation. While the ultra-low parent ratio of titratable acid content was higher, showing a trend of low genetic variation.

**Table 10:** Variation analysis of fruit traits in triploid progeny.

Traits		Years	Variation coefficient/%	Variation range	Mid-parent heterosis/%	Ultra-high parent ratio/%	Ultra-low parent ratio/%
fruit size traits	Single fruit weight/g	2022	50.14	3.82~64.34	26.27	27.08	4.17
		2023	46.29	5.12~52.84	16.01	17.65	11.76
	Fruit length/mm	2022	21.82	20.49~59.01	7.01	22.92	8.33
		2023	23.62	18.28~55.82	-10.29	5.88	50.98
	Fruit diameter/mm	2022	17.28	19.14~51.91	12.39	33.33	6.25
		2023	17.95	20.21~44.51	10.28	17.65	3.92
	Fruit shape index	2022	10.31	0.72~1.14	5.43	54.17	18.75
		2023	12.38	0.77~1.38	26.51	74.51	0.00
	Edible rate/%	2022	1.00	94.09~98.74	1.80	72.34	4.26
		2023	1.40	92.36~99.10	-0.43	1.89	18.87
fruit kernel traits	Single fruit kernel weight/g	2022	50.00	0.21~1.59	-18.42	10.64	44.68
		2023	52.73	0.20~1.54	66.67	67.92	9.43
	Kernel longitudinal diameter/mm	2022	22.71	12.00~31.58	-2.38	27.66	42.55
		2023	24.27	11.52~33.44	-0.93	18.87	30.19
	Kenel transverse diameter/mm	2022	14.88	6.91~13.15	3.20	25.53	12.77
		2023	16.81	6.16~14.39	22.43	58.49	1.89
	Kernel index	2022	16.59	1.56~3.29	-5.11	21.28	48.94
		2023	18.78	1.34~2.92	-19.59	9.43	88.68
fruit nutritional traits	Vitamin C content/(mg/100g)	2022	14.19	207.20~387.39	-9.07	22.92	72.92
		2023	20.57	285.46~633.55	36.99	72.73	0.00
	Soluble solid content/%	2022	11.30	23.93~38.00	-12.38	0.00	41.67
		2023	10.50	30.03~46.15	0.83	14.55	14.55
	Soluble sugar content/%	2022	22.13	17.87~40.50	-3.15	21.28	14.89
		2023	9.79	19.22~33.01	2.56	14.55	34.55
	Titratable acid content/%	2022	18.00	0.31~0.76	-12.28	70.21	17.02
		2023	16.39	0.42~0.92	15.09	9.09	61.82

### 3.4 Screening of large-fruit and high-sugar triploid lines

All the triploid progeny were ordered according to the single fruit weight and soluble sugar content from high to low, and the top 3 large-fruit triploid superior lines and the top 3 high-sugar triploid superior lines were selected from the triploid progeny (**Table 11**). The single fruit weight of T11 was 52.84 g, which was about 20 g than that of the male parent. The soluble sugar content of T167 was 33.01%, which was about 10% higher than that of the parents.

**Table 11:** Superior lines of large-fruit and high-sugar in triploid progeny.

Type	No.	Single fruit weight/g	Fruit Length /mm	Fruit diameter /mm	Fruit shape index	Vitamin C content /(mg/100g)	Soluble solid Content/%	Soluble sugar Content/%	Titratable acid Content/%
Large fruit	T11	52.84	55.82	43.10	0.77	36.07	508.82	22.13	0.63
	T156	49.33	51.29	41.98	0.82	42.14	471.68	24.46	0.58
	T180	47.88	50.54	44.51	0.90	35.44	308.94	25.69	0.56
High sugar	T167	23.55	35.48	32.05	0.91	46.15	396.51	33.01	0.49
	T181	6.97	21.46	23.10	1.08	36.85	506.56	26.84	0.82
	T16	13.48	26.97	29.93	1.11	40.45	435.54	26.69	0.54

## 4 Discussion

### 4.1 The occurrence of polyploid hybrid progeny

Meiosis is an important way of cell division, and its normal operation is of great significance to the genetic stability of organisms. However, abnormal meiosis behavior can lead to abnormalities in the number or structure of chromosomes in gametes, and can also affect pollen size and fertility. The male parent ‘Chenguang’ occurs abnormal behavior in the process of meiosis. The chromosomal configuration is complex at meiotic diakinesis, with univalents, bivalents, trivalents, quadrivalents, and two nucleoli. Anaphase I and anaphase II showed the phenomenon of abnormal cytokinesis. Diad, triad, polyad and some micronucleus cells also appeared in the tetrad period (Lv et al., 2018). We measured the size of ‘Chenguang’ pollen and found that the proportion of normal pollen (2x) was 67.33%, the proportion of small pollen (x) and large pollen (3x) was 30% and 2.67%, respectively (to be published). We speculated that the pollen size and fertility of ‘Chenguang’ were affected by the abnormal behavior at different periods of meiosis, resulting in the emergence of diploid progeny. However, the occurrence of diploid remains unclear, and further research is needed. Through sexual polyploidy hybridization, we created a batch of allotriploid germplasm. These materials have the advantages of hybridization and ploidy effect, the gene heterozygosity is higher, and the genome impact phenomenon makes them more abundant phenotype and biological traits variation, which is more suitable for plant genetic improvement.

### 4.2 Triploid hybrid progeny of jujube has typical polyploid traits

After sexual polyploidization, the biological characteristics of most fruit trees changed due to chromosome doubling, such as strong growth, thick branches, large leaves, big fruits and seedless, and these traits showed high genetic stability in the process of reproduction (Liu et al., 2011). Qiu (2022) found that, the leaf length of jujube polyploid progeny became shorter, the leaf width became wider, and the leaf shape index became smaller, indicating that the leaf of jujube polyploid progeny was rounder. This study found that the progeny of jujube triploid had polyploid characteristics such as shorter leaf length, wider leaf width and smaller leaf shape index. Wei et al. (2022) found that the leaf length and leaf shape index of cassava tetraploid were smaller than that of diploid, while the leaf width was larger than that of diploid, which was consistent with our research results. The outcomes demonstrated by other researchers have shown that stomatal size and stomatal density have a certain correlation with plant resistance (Franks et al., 2015). As stomatal density decreases, the plant resistance tends to increase (Ramos and Volin, 1987). Dang et al. (2011) showed that apple varieties with smaller stomatal density had stronger resistance to brown spot disease. Zhao et al. (2017) found that the stomatal density of pear varieties resistant to black star disease was lower than that of pear varieties susceptible to black star disease. In this study, the stomatal density of jujube triploid was lower than that of diploid, indicating that jujube triploid had strong stress resistance. Liu et al. (2024) found that the stomatal length and width of tetraploid Cyclocarya paliurus were larger than that of diploid, and the stomatal density was smaller than that of diploid. In addition, tetraploid Fortunella hindsii Swingle (Zhang et al., 2022) have similar stomatal characteristics. This study investigated and analyzed characters of jujube triploid offspring for the first time, found that the stomatal length and stomatal width of the triploid offsprings of jujube became larger, and the stomatal density became smaller, which refers to the characteristics of typical polyploid. It also showed that the triploid progeny of jujube had strong resistance from a biological point of view. The above-mentioned characteristics of leaves and stomatal traits in triploid progeny of jujube were in line with the characteristics of polyploidy, which can be used as the most direct and effective method for preliminary identification of ploidy of hybrid offspring of jujube, and it will greatly reduce the workload and difficulty during jujube breeding process.

After polyploidy of fruit trees, different characters of different species will also show some differences (Abdolinejad et al., 2021). In terms of fruit size, polyploid fruits are generally larger than diploid ones. Wang et al. (2022) found that the single fruit weight, longitudinal diameter and transverse diameter of apple triploid fruits all increased. Liu et al. (2022) found that the single fruit weight, transverse diameter, longitudinal diameter and edible rate of jujube tetraploid increased. However, in the pear tetraploid (Wang et al., 2015), the fruit does not increase, and its growth rate is higher than that of the diploid. In this study, it was found that single fruit weight, transverse diameter, longitudinal diameter, fruit shape index and edible rate of jujube triploid increased, indicating that the offspring of jujube triploid fruit had typical characteristics of large fruit. Studies have found that a single plant with high sugar and low acid appeared in the hybrid progeny of apple (Huang et al., 2024) and apricot (Jiang et al., 2018), while a single plant with high sugar and high acid appeared in the hybrid offspring of grape (Jia et al., 2021). This study found that the titratable acid content of the fruit of jujube triploid progeny was lower, the soluble solid content and soluble sugar content were higher, and the phenomenon of high sugar and low acid appeared. We speculate that this may be related to the genetic characteristics of the parents, or it may be related to environmental factors. It can be seen that with the increase of ploidy, the fruit quality of jujube triploid progeny has been improved, and resources with excellent quality traits can be selected to provide important materials for new variety breeding.

### 4.3 Abondance of genetic variations of traits in triploid progeny

As the main vegetative organ of fruit trees, leaves play an important role in the growth and development of fruit trees. Liu et al. (2023) Studied the leaf phenotypic traits of F1 generation in Eucommia ulmoides showed that the coefficient of variation ranged from 6.29% to 36.97%. Among them, the variation range of leaf area was higher, the coefficient of variation was 36.97%. In addition, the study on F1 generation of persimmon (Diao et al., 2017)showed that the coefficient of variation of leaf phenotypic traits ranged from 6.87 to 42.78%, and the mid-parent heterosis ratio of 11 traits was negative. This study found that the coefficient of variation of leaf traits in triploid progeny was 13.13~33.35%. Among them, the variation range of leaf area was higher, the coefficient of variation was 33.35%, while the variation range of leaf shape index was smaller, the coefficient of variation was 13.13%, and the mid-parent heterosis ratio is negative. The results showed that the leaf traits had heterosis with dominant genetic effects and that jujube trees had high heterozygosity, which was basically consistent with previous studies on leaf variation of apple (Dang et al., 2012) and jujube (Qiu et al., 2023). Thorns is a special character of jujube, which mainly plays the role of self-protection. In production practice, it is easy to pick jujube fruit without or with few thorns. Qi et al. (2009) found that the variation coefficient of thorn length ranged from 23.53% to 27.86%, and the variation of traits among individuals was large, while the difference between traits was small. Lu et al. (2003) found that the variation coefficient of thorn traits of the naturally pollinated seedlings of different varieties varied greatly. In this study, the variation coefficient of thorn traits of jujube triploid progeny ranged from 16.95% to 34.29%, and showed a trend of large genetic variation, the heterosis and super high parent advantage was noticeable. The variation of leaf and thorn traits of triploid progeny was abundant, and it might be due to the highly heterozygous nature of jujube trees, non-additive effect of the gene was recombined during the hybridization process and ploidy effect.

Stomata is an important organ for gas and water exchange between plants and the external environment, and playing a crucial role in plants physiological processes. In the study of Dong (2016), the stomatal density, length and width of the ‘Qinguan’×’Honeycrisp’ hybrid showed varying degrees of variation, and the phenomenon of bidirectional superparental separation was presented. In this study, the genetic variation of stomatal traits in jujube triploid offspring was analyzed. It was found that the coefficient of variation of stomatal traits in triploid hybrid progeny was 11.96~15.18%, indicating rich genetic diversity and great selection potential. The mid-parent heterosis rate of stomatal traits in triploid progeny was -13.82~1.35%, and the genetic transmission ability was 86.18~101.35%. The genetic tendency of stomatal width and stomatal density showed a small genetic trend, but the genetic tendency of stomatal length showed a large genetic trend, and the heterosis was obvious. Shang et al. (2020) found that the variation of stomatal traits of Populus cathayana was mainly due to ploidy effect, followed by genotype effect and environmental effect. Bai et al. (2015) found that ploidy had a great impact on leaf, stomata and other characters of Populus tomentosa. In this study, compared with the diploid offspring, the leaves and stomata of the triploid progeny in jujube were significantly larger. It is speculated that the main reason for the variation of stomatal traits in triploid progeny of jujube is ploidy effect, and the secondary reason is hybridization effect.

Fruit quality is an important indicator to measure whether fruit tree varieties are excellent, and it also directly affects the consumers’ willingness to purchase, thereby improving their economic value. Genetic improvement of fruit size and shape can be achieved by sexual polyploidization. Han et al. (2022) found that the variation coefficient of single fruit weight of kiwi hybrid offspring was above 20% for three consecutive years, the genetic transmission ability was lower than 100%, and the mid-parent heterosis rate was negative, indicating that single fruit weight was susceptible to environmental influences and heterosis was not obvious. Wu et al. (2022) studied the fruit quantitative traits of 116 jujube germplasm resources and found that the variation coefficient of single fruit quality was the largest (41.30%) and the variation coefficient of edible rate was the smallest (1.76%). In this study, the diversity of fruit size traits of triploid progeny of jujube was analyzed. The variation coefficient of single fruit weight was the largest (50.14%) and the variation coefficient of edible rate was the smallest (1%), indicating that single fruit weight was widely separated, the variation degree was large, and the influence of genetic regulation and environment was great, and the genetic improvement potential was great. This is consistent with the results of Pan et al. (2023) on fruit size traits of jujube. The variation coefficient of fruit size traits in this study was generally higher than that in previous studies, indicating that the genetic diversity of fruit size in triploid progeny was richer, which may be related to different test materials or ploidy hybridization. Yang et al. (2023) found that the fruit appearance heredity of the hybrid offspring of Jujube and Wild Jujube was more biased to the father, while the fruit nutrition index heredity was more biased to the mother. In this study, the genetic transmission ability of fruit traits in jujube triploid offspring was higher than 80%, showed a large genetic trend, obvious heterosis, and fruit size traits were more biased to the father, which may be due to gene recombination in the process of sexual polyploidy, resulting in the appearance of ‘Chenguang’ large fruit traits. Therefore, when breeding large fruit type progeny as the breeding objectives, we should choose large fruit varieties as parents as much as possible, in order to obtain more large fruit type progeny. Due to the unusual situations in 2022, the fruit picking was delayed, which may have impacted the determination of fruit nutritional traits, mainly measured in 2023. In this study, the variation analysis of fruit nutritional traits of jujube triploid progeny was analyzed, and the variation coefficient of vitamin C content was the largest (20.57%), and showed ultra-high parent heritability, indicating greater genetic potential and selection space in triploid progeny, which is consistent with the research results of Xie et al. (2022) on the content of vitamin C in ‘Dongzao’ × ‘Jinsi 4’ hybrid F1 generation. On the contrary, the variation coefficient of vitamin C content in strawberry Wang and Wang (2023) hybrids was the lowest, the degree of variation was small, and the trait inheritance was more stable. Therefore, we inferred that the genetic variation of vitamin C content is mainly additive effect, and there is a positive non-additive effect.

The biological traits of polyploid progeny obtained by sexual polyploidization are usually affected by both ploidy and hybridization effect. Ploidy effect is an important factor affecting the variation of sexual offspring traits. Liu et al. (2024) found that ploidy variation had an effect on leaf size, shape, branch diameter and internode length of ‘Cuimi Kumquat’. Zhang et al. (2017) found that with the increase of ploidy, the leaf thickness, upper epidermis, palisade tissue and sponge tissue in rubber trees indicated significantly differences. This study found that the leaf and fruit traits of different ploidy progeny were significantly different, and the variation degree of triploid progeny was greater than that of diploid progeny, indicating that ploidy effect had a certain degree of influence on progeny traits. Hybridization effect is the premise of the variation of progeny traits. Li et al. (2023) studied the genetic diversity of two different jujube hybrid offspring groups and natural pollination progeny groups, and found that the genetic diversity levels of the three groups were significantly different. Ma et al. (2024) studied the variation of long branch and leaf traits of diploid and triploid hybrids of Populus cathayana. It was found that leaf traits such as leaf length, leaf width, leaf area and petiole length were mainly affected by genotype effect, and stomatal length, stomatal width and stomatal density were mainly affected by ploidy effect. The comprehensive analysis of this study found that the phenotypic traits such as leaf size, stomatal size, and fruit size of the triploid progeny of jujube were more susceptible to ploidy effects, while the internal quality of the fruit was affected by ploidy and hybridization effects.

Polyploidy is an important driving force for plant evolution. After polyploidy, there are corresponding changes in chromosome number and chromosome recombination in the genome, and epigenetic changes in gene expression, such as gene silencing and activation, gene non-additive expression, sequence elimination, transposon and DNA methyl pattern, small molecule RNA, and nucleolar dominance, etc. Many genes and gene families also exhibit copy number changes after multiploidy. It was found that genome hybridization had more effect on gene expression than genome doubling. Based on genomic evidence, Feng et al. (2024) proposed a framework model of the polyploidization-rediploidization process to reveal the mechanism of plant genome ploidy change and adaptive evolution in the context of global climate change. Blasio et al. (2022) proposed that genomic changes and transcriptome modifications produced in primitive allopolyploids can add complexity during evolution, resulting in phenotypic innovations. Harun et al. (2024) found that If polyploid plants are stable effectively, they provide advantages over their diploid counterparts. Polyploid has higher biomass, yield, vigor, larger vegetative organs, seeds, and seed pods compared to diploid. Therefore, allopolyploids are more complex in terms of genetic mechanism and molecular regulation, and their character variation is more obvious.

## 5 Conclusion

This study indicate that the triploid progeny exhibit typical polyploid characteristics, such as round leaves and large stomata, which can serve as preliminary indicators for identifying the ploidy of hybrid progeny. The fruit size of triploid offspring demonstrates a prominent trait of larger fruits, while their nutritional quality exhibits distinct features of high sugar content and low acidity, thus indicating excellent fruit quality traits in the progeny. The leaf structure, stomatal characteristics, and fruit size in triploid jujube progeny are significantly influenced by ploidy effects; however, both ploidy effects and hybridization effects impact the fruit quality. The allotriploid germplasm of jujube showed the advantages of huge fruit and high metabolic content, which provided new material for breeding and genetic research of jujube cultivars in the future, and also provided a method reference for ploidy hybridization of other varieties and tree species.

## Data Availability

The original contributions presented in the study are included in the article/**Supplementary Material**. Further inquiries can be directed to the corresponding author.

## References

[B1] Abdolinejad R. Shekafandeh A. Jowkar A. (2021). *In vitro* tetraploidy induction creates enhancements in morphological, physiological and phytochemical characteristics in the fig tree (Ficus carica L.). Plant Physiol. Biochem. 166, 191–202. doi: 10.1016/j.plaphy.2021.05.047 34118682

[B2] Bai F. Y. Zeng Q. Q. Kang N. Suo Y. J. Liao T. T. Zhang P. D.. (2015). Ploidy level and contrast analysis of the traits for superior trees of Populus tomentosa Carr. in gene pool. J. Beijing forestry university. 37, 113–119. doi: 10.13332/J.1000-1522.20140247

[B3] Blasio F. Prieto P. Pradillo M. Naranjo T. (2022). Genomic and meiotic changes accompanying polyploidization. Plants (Basel Switzerland). 11, 125. doi: 10.3390/plants11010125 35009128 PMC8747196

[B4] Chen S. S. He Y. Q. Xu X. B. Jia D. F. Tao J. J. Mei Y. Y.. (2021). Effect of different harvest time on fruit quality of ‘Fenghuang No.1’kiwi fruit. Acta agriculturae universitatis Jiangxiensis. 43, 1259–1268. doi: 10.13836/j.jjau.2021135

[B5] Dang Z. G. Gao H. Wang L. C. Lv Y. M. Zhao Z. Y. (2011). Evaluation and physiological analysis of resistance of apple (Malus domestica Borkh.) cultivars to apple brown spot [Marssonina Mali (P. Henn.) Ito. Plant Physiol. J. 47, 691–698. doi: 10.13592/j.cnki.ppj.2011.07.004

[B6] Dang W. F. Zhang J. K. Wang T. Wen B. N. (2011). Genetic analysis on biological characters of Qinguan × fuji apple in F1 population. J. Henan Agric. Sci. 41, 122–126. doi: 10.15933/j.cnki.1004-3268.2012.10.032

[B7] Diao S. F. Li F. D. Duan W. Han W. J. Sun P. Fu J. M. (2017). Genetic diversity of phenotypic traits of leaves in F1 progeny of persimmon. J. China Agric. university. 22, 32–44. doi: 10.11841/j.issn.1007-4333.2017.0204

[B8] Dong L. (2016). The analysis of stomatal traits and QTL mapping under drought condition in ‘Qinguan’ × ‘Honeycrisp’ leaves (Yangling: Northwest Agriculture and Forestry University).

[B9] Du K. (2021). Epigenetic regulation mechanism of vegetative growth advantage in allotriploid Populus spp. (Section Tacamahaca) (Beijing: Beijing Forestry University).

[B10] Feng X. Chen Q. P. Wu W. H. Wang J. X. Li G. H. Xu S. H.. (2024). Genomic evidence for rediploidization and adaptive evolution following the whole-genome triplication. Nat. Commun. 15, 1635. doi: 10.1038/s41467-024-46080-7 38388712 PMC10884412

[B11] Franks P. J. Doheny-Adams T. W. Britton-Harper Z. J. Gray J. E. (2015). Increasing water-use efficiency directly through genetic manipulation of stomatal density. New phytologist. 207, 188–195. doi: 10.1111/nph.2015.207 25754246

[B12] Gao Q. C. (2011). Identification of agronomic and biological characters of being introduced grape varieties forjuicing from USA. (dissertation) (Yangling: Northwest Agriculture & Forestry University).

[B13] Guan S. P. Wang T. T. Zhou Y. G. Zhu H. X. Wu X. M. Long C. R.. (2024). Inheritance of some quality traits of the fruits in triploid hybrids derived from two citrus 2x × 4x interploidy crosses. J. fruits science. 41, 369–378. doi: 10.13925/j.cnki.gsxb.20230419

[B14] Guo H. J. Jin X. W. Shen C. J. Liu Z. X. Li B. B. Yang H.. (2023). Present situation and outlook of Xinjiang red jujube industry. J. Huazhong Agric. university. 42, 35–41. doi: 10.13300/j.cnki.hnlkxb.2023.05.005

[B15] Han F. Zhao T. T. Liu X. L. Zhang Q. Li D. W. Tian H.. (2022). Genetic analysis of fruit traits in Actinidia rufa (Siebold and Zuccarini) Planchon ex Miquel × Actinidia chinensis var. chinensis C. F. Liang kiwifruit hybrid population. Plant Sci. J. 40, 505–512. doi: 10.11913/PSJ.2095-0837.2022.40505

[B16] Harun A. Fang Z. Chen C. (2024). The contributions of cytogenetics, genetics, and epigenetics to the stability of plants polyploidy. *Discov* . Plants 1, 11. doi: 10.1007/s44372-024-00012-3

[B17] He P. Wang Y. X. Li L. G. Wang H. B. Li H. F. Sun Q. R.. (2015). Genetic analysis of apple triploid hybrid progenies from diploid and tetraploid based on ISSR. Shandong Agric. Sci. 47, 11–13. doi: 10.14083/j.issn.1001-4942.2015.09.003

[B18] Huang J. F. Lu J. X. Yan Z. Y. Wang D. M. Yang F. Wang Y. D.. (2024). Analysis of genetic variation of sugar and acid contents in F1 population of apple derived from ‘Changfu No.2’×’Jinhong’. Special economic Anim. plants. 27, 9–43.

[B19] Jia N. Li M. M. Han B. Yin Y. G. Liu C. J. Sun Y.. (2021). Analysis of genetic predispositions for fruit sugar-acid traits in the progeny of a cross between ‘Rosette’ and ‘Red Earth’ grapes. Hebei fruits. 4, 9–10 + 12. doi: 10.19440/j.cnki. 1006-9402.2021.04.004

[B20] Jiang F. C. Sun H. Y. Yang L. Zhang J. H. Wang Y. Z. (2018). Analysis of genetic variation of sugar and acid contents in F1 population of apricot derived from ‘Chuanzhihong’ × ‘Luotuohuang’. J. fruits science. 35, 649–657. doi: 10.13925/j.cnki.gsxb.20170341

[B21] Li M. Ge W. J. Shen J. Liu S. H. (2021). Effect of adding rice husk ash to the cultivation substrate on the growth and fruit quality of melon. Acta bot.boreal.-occident.sin. 41, 1736–1746. doi: 10.7606/j.issn.1000-4025.2021.10.1736

[B22] Li M. Y. Shi G. C. Zhu J. R. Xie H. Li X. G. (2021). Comprehensive evaluation of fruit quality of fresh jujube Dongzao. Non-wood For. Res. 39, 256–263. doi: 10.7606/j.issn.1000-4025.2021.10.1736

[B23] Li B. Y. Wei T. J. (2013). Research progress of jujube breeding technology. Ningxia J. Agric. fores. sci.&tech. 54, 27–29 + 53.

[B24] Li F. Zhen M. Yang S. Shang G. L. Wang J. R. Liu M. J. (2023). Analysis of genetic diversity and leaf trait variation in Chinese Jujube offspring. Mol. Plant breeding.

[B25] Liu M. J. (2010). Chinese jujube: botany and horticulture. Hortic. review. 32, 229–298. doi: 10.1002/9780470767986.ch5

[B26] Liu X. S. Chen L. Wang J. X. Li L. Peng J. Y. (2013). Discovery and identification of natural triploid ploidy of Chinese Jujube cultivar’Pingguozao’. Acta Hortic. sinica. 40, 426–432. doi: 10.16420/j.issn.0513-353x.2013.03.004

[B27] Liu X. H. Cui S. Q. Liu N. N. Xu G. (2011). Research Progress in polyploid breeding of jujube. Hunan Agric. science. 18, 6–18 + 30. doi: 10.16498/j.cnki.hnnykx.2011.18.021

[B28] Liu T. J. Ding J. Zhao S. H. Zhang Q. Zhang G. Q. Li Y. C.. (2013). Thoughts and practices on crossbreeding of jujube tree. Ningxia J. Agric. fores. sci.&tech. 54, 17–18.

[B29] Liu B. H. Ge J. Deng G. Z. Tang Z. P. Wu Q. Q. Deng Z. N. (2024). Identification of ploidy of ‘Crisp Honey Kumquat’ and comparison of morphological traits of different ploidy of Kumquat branches and leaves. South. horticulture. 35, 06–14.

[B30] Liu M. J. Liu P. Jiang H. E. Wu G. E. Liu Z. G. (2010). A new tetraploidy table Chinese Jujube cultivar ‘Chenguang’. Acta Hortic. sinica. 37, 1539–1540. doi: 10.16420/j.issn.0513-353x.2010.09.024

[B31] Liu J. X. Liu P. Minavar Y. Wang J. R. Liu M. J. Yan F. F. (2022). Comparison of phenotypic characters between diploid and autotetraploid of Chinese Jujube in southern Xijiang. Acta agriculturae boreali -occidentalis sinica. 1, 595–602. doi: 10.7606/j.issn.1004-1389.2022.05.008

[B32] Liu X. L. Song Z. Q. Hu F. R. Shang X. F. (2024). A comparative study on leaf characters between diploid and tetraploid of Cyclocarya paliurus. J. Nanjing forestry Univ. (Natural Sci. edition). 48, 76–84. doi: 10.12302./j.issn.1000-2006.2023.05.024

[B33] Liu M. J. Wang M. (2009). Chinese jujube germplasm resources (Beijing, China: China forestry press).

[B34] Liu C. L. Zhong J. Qing J. Wang L. Du H. Y. Liu P. Y.. (2023). Leaf phenotypic variation and heterosis of F1 generation of Eucommia ulmoides. Non-wood For. Res. 41, 225–235. doi: 10.14067/j.cnki.1003-8981.2023.01.023

[B35] Lu Z. M. Liu K. Yan Z. X. Li X. G. (2010). Progress of research on nutrient composition and health effects of jujube fruit. Acta Hortic. sinica. 37, 2017–2024. doi: 10.16420/j.issn.0513-353x.2010.12.006

[B36] Lu J. Y. Mao Y. M. Shen L. Y. Peng S. Q. Li P. L. Ma Q. H. (2003). Study on the trait segregation of Chinese jujube naturally pollinated seedlings. J. Agric. Univ. Hebei. 4, 53–58 + 67.

[B37] Lv Y. Xue Z. H. Wu G. E. Liu P. Liu M. J. (2018). Abnormal meiosis behaviors of triploid and tetraploid Chinese Jujube. Acta Hortic. sinica. 45, 659–668. doi: 10.16420/j.issn.0513-353x.2017-0469

[B38] Ma H. W. Ren Y. X. Long Y. X. Wang N. Feng X. Y. Yu T. Q.. (2024). Variation in leaf traits on long branches in full-sib diploid and triploid hybrids between section Tacamahaca and sect. Aigeiros of Populus. J. Beijing forestry university. 46, 27–34. doi: 10.12171/j.1000-1522.20220339

[B39] Mao Y. M. Song R. P. Shen L. Y. (2008). GB/T2U45 Quality grade of fresh jujube (Beijing, China: China standard publication).

[B40] Miller-Rushing A. J. Primack R. B. Templer P. H. Rathbone S. Mukunad S. (2009). Long-term relationships among atmospheric CO2, stomata, and intrinsic water use efficiency in individual trees. Am. J. botany. 96, 1779–1786. doi: 10.3732/ajb.0800410 21622298

[B41] Pan Y. L. Bao J. K. Chen W. N. Wu C. Y. Wang J. R. Liu M. J.. (2023). Genetic analysis of fruit traits and selection of superior lines in F1 generation of jujube JMS2 × Jiaocheng 5. J. fruits science. 40, 1085–1098. doi: 10.13925/j.cnki.gsxb.20220602

[B42] Peng J. Y. Liu P. Zhou J. Y. Peng S. Q. Cao Q. G. Chu X. F. (2005). Karyotypes of different strains in Ziziphus jujuba Mill. ‘Zanhuang Dazao’. Acta Hortic. Sin. 5, 798–801. doi: 10.16420/j.issn.0513-353x.2005.05.008

[B43] Pu F. S. (1990). Fruit Tree Germplasm Resource Descriptors (Beijing, China: China forestry press).

[B44] Pu T. T. Zhang Q. Wang Y. R. Sun P. Bai Y. E. Suo Y. J.. (2013). Research advances and prospects in polyploid breeding of persimmon. J. Fruits Sci. 40, 1741–1749. doi: 10.13925/j.cnki.gsxb.20220655

[B45] Qi J. Dong Z. Shen L. Y. Mao Y. M. Li Y. H. Liu J.. (2009). Analysis of QTL for needle length in Chinese Jujube. Acta Hortic. sinica. 36, 807–813. doi: 10.16420/j.issn.0513-353X.2009.06.007

[B46] Qi C. F. Wang Q. F. Niu Y. H. Zhang Y. Liu M. J. Liu Z. G.. (2024). Characteristics of ZjCIPKs and ZjbHLH74-ZjCIPK5 regulated cold tolerance in jujube. Int. J. Biol. macromolecules. 264, 130429. doi: 10.1016/j.ijbiomac.2024.130429 38428762

[B47] Qiu K. (2022). Ploidy identification and character segregation of hybrid progenies of a cross between diploid ‘Dong zao’ and tetraploid ‘Chen guang’ in Chinese Jujube. (dissertation). (Alar: Tarim University).

[B48] Qiu Q. Q. Feng Y. F. Wu C. Y. (2021). Analysis of genetic diversity of leaf phenotypic traits in jujube germplasm resources. Xinjiang Agric. Sci. 58, 282–293. doi: 10.6048/j.issn.1001-4330.2021.02.010

[B49] Qiu K. Pan Y. L. Bao J. K. Lin M. J. Wu C. Y. Wang J. R.. (2022). Controlled cross-breeding technology system for jujubes on a large scale. Hebei Fruits. 1, 1–3. doi: 10.19440/j.cnki.1006-9402.2022.01.001

[B50] Qiu Q. Q. Xia Y. L. Bao J. K. Yan M. Yang Z. Wang C. C.. (2023). Analysis of the genetic law of Jujube hybrid F1 generation based on leaf phenotypic traits. Mol. Plant breeding. 21, 7456–7465. doi: 10.13271 j.mpb.021.007456

[B51] Ramos L. J. Volin R. B. (1987). Role of stomatal opening and frequency on infection of Lycopersicon spp. by Xanthomonas campestris pv. vesicatoria. Phytopathology 77, 311–1317. doi: 10.1094/PHYTO-77-1311

[B52] Shang J. Xue Y. X. Song L. J. Liu C. H. Li D. L. Zhang H. Y.. (2020). Ploidy, genotype and gender effects of functional leaf and stomatal traits on short branches in fullsib hybrids between section Tacamahaca and sect. Aigeiros of Populus. J. Beijing forestry university. 42, 11–18. doi: 10.12171/j.1000-1522.20200095

[B53] Wang F. Fang C. Q. Jiang S. L. Lin S. H. Ou C. Q. Li L. W.. (2014). A new triploid pear cultivar ‘Huaxing’. Acta Hortic. sinica. 41, 2355–2356. doi: 10.16420/j.issn.0513-353x.2014.04.004

[B54] Wang F. Jiang S. L. Chen Q. J. Ou Q. C. Zhang W. J. Hao N. N.. (2016). Changes in fruit texture of crisp-flesh pear during fruit ripening. J. Fruit science. 33, 950–958. doi: 10.13925/j.cnki.gsxb.20160008

[B55] Wang L. X. Liu Z. G. Han S. K. Liu P. Sadeghnezhad E. Liu M. J. (2023). Growth or survival: What is the role of calmodulin-like proteins in plant? Int. J. Biol. macromolecules. 242, 124733. doi: 10.1016/j.ijbiomac.2023.124733 37148925

[B56] Wang L. H. Lu Y. Q. Su H. Zhang Q. Zhao Z. J. Chen J. Y.. (2022). Research progress on polyploidy breeding of fruit trees. J. Shanxi Agric. university(Natural Sci. edition). 42, 14–24. doi: 10.13842/j.cnki.issn1671-8151.202203047

[B57] Wang L. H. Lv Y. Luo Z. Liu P. Liu M. J. (2018). Establishment and application of a method for chromosome ploidy identification and genome size estimation using flow cytometry in Ziziphus jujuba. J. Agric. Biotechnol. 26, 511–520. doi: 10.3969/j.issn.1674-7968.2018.03.01

[B58] Wang B. M. Nie J. L. Su Y. Pei Y. Qian Y. Y. Liu H. R. (2021). Introduction experiment of ‘Qiuping’ red raspberry in Tianjin. Non-wood For. Res. 39, 227–234. doi: 10.14067/j.cnki.1003-8981.2021.03.026

[B59] Wang S. Y. Shi X. X. Yang L. L. Chu F. J. Du G. Q. (2014). Embryo rescue for hybrid after crossing of seedless diploid with tetraploid and analysis of its progeny ploidy. J. Agric. Univ. Hebei. 37, 55–60. doi: 10.13320/j.cnki.jauh.2014.011

[B60] Wang Y. Wang L. J. (2023). Genetic analysis of fruit traits in Strawberry hybrid offspring. Mol. Plant breeding.

[B61] Wang X. Q. Wang H. H. Shi C. H. Zhang X. Y. Duan K. Luo J. (2015). Morphological, cytological and fertility consequences of a spontaneous tetraploid of the diploid pear (Pyrus pyrifolia Nakai) cultivar ‘Cuiguan’. Scientia horticulturae. 189, 59–65. doi: 10.1016/j.scienta.2015.03.048

[B62] Wang T. K. Zhang J. Z. Qi Y. S. Pang H. Z. (2004). Research progress on polyploid breeding of fruit trees in China. J. fruits science. 6, 592–597. doi: 10.13925/j.cnki.gsxb.2004.06.020

[B63] Wang T. T. Zhou Y. G. Zhu H. X. Zhang M. Duan Y. W. Cao H. X.. (2022). Inheritance of sugar and acid contents in the fruits of triploid hybrids originated from two 2x × 4x crosses with Nadorcott tangor as a female parent. J. fruits science. 39, 1147–1156. doi: 10.13925/j.cnki.gsxb.20210674

[B64] Wei W. L. Huang Z. L. Chen H. X. Yang H. X. Lan X. Liang Z. H.. (2022). Comparison of morphological, physiological and mite resistance characteristics of cassava diploid and its autotetraploid leaves. J. Nucl. Agric. Sci. 36, 2115–2123. doi: 11869./j.issn.100-8551.2022.11.2115

[B65] Wu C. Y. Chang H. W. Lin M. J. Cai G. X. Yu J. (2016). Xinjiang jujube industry development status quo and its problems. Northern fruits. 6, 41–44. doi: 10.16376/j.cnki.bfgs.2016.06.021

[B66] Wu H. Su W. L. Shi M. J. Xue X. F. Ren H. Y. Wang Y. K.. (2022). Diversity analysis and comprehensive evaluation of Jujube fruit traits. J. Plant Genet. resources. 23, 1613–1625. doi: 10.13430/j.cnki.jpgr.20220417001

[B67] Xiang X. D. Gao Y. K. Cui J. H. Ren G. Z. Wei S. L. Zakeyeldinn E. A.. (2019). Genetic variation analysis in F2 population from autotetraploid grape hybrid. J. Plant Genet. Resources. 20, 960–974. doi: 10.13430/j.cnki.jpgr.20181204003

[B68] Xiao Y. (2013). Genetic Diversity and Population Structure of Chinese Jujube Analyzed by SSR Markers. (dissertation). (Yangling: Northwest Agriculture & Forestry University).

[B69] Xiao J. (2014). Characterization of SSR loci and primer development for jujube genome. (dissertation). (Hebei: Agricultural University of Hebei).

[B70] Xie H. Wang Z. T. Li M. Y. Li X. G. (2022). Genetic analysis of fruit characters in hybrid progeny of Chinese jujube. Non-wood For. Res. 40, 125–134. doi: 10.14.67/j.cnki.1003-8981.2022.02.013

[B71] Xie K. D. Wang X. P. Wang H. Q. Liang W. J. Xie Z. Z. Guo D. Y.. (2014). High efficient and extensive production of triploid Citrus plants by crossing polyembryonic diploids with tetraploids. Acta Hortic. Sinica. 41, 613–620. doi: 10.16420/j.issn.0513-353x.2014.04.004

[B72] Xie K. D. Wang H. Q. Wang X. P. Liang W. Xie Z. Z. Yi H. L.. (2013). Extensive citrus triploid breeding by crossing monoembryonic diploid females with allotetraploid male parents. Scientia Agricultura Sinica. 46, 4550–4557. doi: 10.3864/j.issn.0578-1752.2013.21.018

[B73] Yan F. F. Wang L. H. Zheng X. J. Luo Z. Wang J. R. Liu M. J. (2018). Acquisition of triploid germplasms by controlled hybridization between diploid and tetraploid in Chinese jujube. J. Hortic. Sci. Biotechnol. 94, 123–129. doi: 10.1080/14620316.2018.1454266

[B74] Yang Z. Zhang C. J. Yang X. F. Dong M. Y. Wang Z. L. Wu C. Y.. (2023). Analysis of fruit genetic tendency and mixed inheritance in hybrid progeny of Jujube and Wild Jujube. Acta Hortic. sinica. 50, 36–52. doi: 10.16420/j.issn.0513-353x.2021-0470

[B75] Yu T. Z. (2022). Analysis on genetic characteristics of hybrid offsprings of Gala apple. (dissertation). (Jinlin: Agricultural University of Jinlin).

[B76] Zhang Y. Y. Guo H. Yuan H. Z. Wang X. J. Huang X. Li Y.. (2017). Stomatal differences among diploid, triploid and tetraploid in Rubber tree. Chin. J. Trop. crops. 38, 389–394. doi: 10.3969/j.issn.1000-2561.2017.03.001

[B77] Zhang C. L. Zhou R. Xie S. P. Shang X. L. (2022). Exploration and evaluation of morphological traits and primary metabolites of tetraploid seedlings from Hongkong kumquat (Fortunella hindsii Swingle). Plant Physiol. J. 40, 47–53. doi: 10.11913/.PSJ.2095-0837.2022.10047

[B78] Zhao J. Wang Y. Chai C. B. Song Y. Q. Li L. L. (2017). Relationship between early structural characteristics of pear leaf and occurrence of scab. J. Shanxi Agric. university(Natural Sci. edition). 37, 575–579. doi: 10.13842/j.cnki.issn1671-8151.2017.08.008

[B79] Zhao X. Zhang Q. T. Fu Y. F. Yang C. X. Zeng L. J. Zhu P.. (2006). Studies on flower characteristic in close plant of sweet potato. J. Southwest China Nor mal Univ. (Natural Science). 31, 143. doi: 10.13718/j.cnki.xsxb.2006.03.033

